# Stimulation of Wnt/ß-Catenin Pathway in Human CD8^+^ T Lymphocytes from Blood and Lung Tumors Leads to a Shared Young/Memory Phenotype

**DOI:** 10.1371/journal.pone.0041074

**Published:** 2012-07-30

**Authors:** Marie-Andrée Forget, Yannick Huon, Alexandre Reuben, Cécile Grange, Moïshe Liberman, Jocelyne Martin, Anne-Marie Mes-Masson, Nathalie Arbour, Réjean Lapointe

**Affiliations:** 1 Research Centre, Centre Hospitalier de l’Université de Montréal (CRCHUM), Université de Montréal and Institut du Cancer de Montréal, Hôpital Notre-Dame, Montréal, Québec, Canada; 2 Division of Thoracic Surgery, Department of Surgery, Université de Montréal, Centre Hospitalier de l’Université de Montréal (CHUM), Montréal, Québec, Canada; National Cancer Institute (INCA), Brazil

## Abstract

Cancer can be treated by adoptive cell transfer (ACT) of T lymphocytes. However, how to optimally raise human T cells to a differentiation state allowing the best persistence in ACT is a challenge. It is possible to differentiate mouse CD8^+^ T cells towards stem cell-like memory (T_SCM_) phenotype upon TCR stimulation with Wnt/ß-catenin pathway activation. Here, we evaluated if T_SCM_ can be obtained from human mature CD8^+^ T cells following TCR and Wnt/ß-catenin activation through treatment with the chemical agent 4,6-disubstituted pyrrolopyrimidine (TWS119), which inhibits the glycogen synthase kinase-3β (GSK-3β), key inhibitor of the Wnt pathway. Human CD8^+^ T cells isolated from peripheral blood or tumor-infiltrating lymphocytes (TIL), and treated with TWS119 gave rise to CD62L^+^CD45RA^+^ cells, indicative of early differentiated stage, also expressing CD127 which is normally found on memory cells, and CD133, an hematopoietic stem cell marker. T_SCM_ cells raised from either TIL or blood secreted numerous inflammatory mediators, but in lower amounts than those measured without TWS119. Finally, generated T_SCM_ CD8^+^ T cells expressed elevated Bcl-2 and no detectable caspase-3 activity, suggesting increased persistence. Our data support a role for Wnt/ß-catenin pathway in promoting the T_SCM_ subset in human CD8^+^ T cells from TIL and the periphery, which are relevant for ACT.

## Introduction

Adoptive cell transfer (ACT) of manipulated mature autologous T lymphocytes in stage IV melanoma patients has shown beneficial effects [1], [2]. Although ACT are very promising anti-cancer therapies, there are still obstacles to circumvent such as expanding ACT to other solid cancers, improving anti-tumor efficacy, simplifying cell culture conditions, and importantly, increasing the persistence of transferred cells. Furthermore, long-term persistence is crucial to prevent cancer recurrence and therefore to establish a long-term anti-tumor memory response. Attempts to define optimal CD8^+^ T cell phenotype for ACT have been deployed using different animal models. By comparing antigen-specific central memory CD8^+^ T (T_CM_) and effector memory cells (T_EM_) for ACT, *Berger et al*. demonstrated that CD8^+^ T_CM_ cells could survive and persist longer after transfer and revert to the memory pool in primates [3]. In a mouse model, *Hinrichs et al.* obtained contrasting results by reporting that antigen-specific T cells originating from naive CD8^+^ T cells (T_N_) were more suitable for ACT when compared to T_CM_. These conclusions were drawn based on an improved cytokine response from the T_N_ post-transfer [4]. The persistence of effector CD8^+^ T cells generated from T_N_ was examined in a second study with human CD8^+^ T cells and demonstrated the presence of longer telomeres and expression of CD27, suggesting a less differentiated T cell phenotype [5].

Recently, another CD8^+^ T cell subset called stem cell memory T cells (T_SCM_) was proposed as a candidate for ACT. T_SCM_ were first identified in a mouse model of human graft-versus-host disease [6]. These cells had the capacity of self-renewal and were shown to favor the development of chronic auto-immune responses. T_SCM_ were defined by low levels of CD44, indicative of a naive phenotype, but elevated levels of CD62L, which is also expressed by T_CM_, as well as CD122 and Bcl-2. Surprisingly, T_SCM_ were found to express Sca-1, a stem cell marker [6]. *Gattinoni et al*. later demonstrated that mouse tumor-specific anti-gp100 CD8^+^ T cells treated with 4,6-disubstituted pyrrolopyrimidine (TWS119), a pharmacologic inhibitor of the glycogen synthase kinase 3β (GSK-3β), could generate a T_SCM_ phenotype [7]. When evaluated in ACT in the gp100-expressing B16 melanoma mouse model, TWS119-induced T_SCM_ mediated a better anti-tumor response when compared to T_CM_ or T_EM_, and persisted in greater numbers than T_CM_ one month post-transfer [7]. Induction of this T_SCM_ phenotype was attributed to the activation of the canonical Wnt pathway following treatment with TWS119. The same group recently reported that T_SCM_ naturally arise in humans, however, a link with the Wnt/ß-catenin pathway remains unknown and no stem-cell marker was identified in these cells [8]. Another study by Muralidharan *et al*. on peripheral and cord blood human T cells reported that activation of the Wnt pathway by either TWS119 or addition of Wnt-3a blocks differentiation in effector T cells [9].

Activation of the canonical Wnt pathway leads to the accumulation and translocation of β-catenin to the nucleus, which activates the transcription factors T cell factor (TCF) and lymphoid enhancer-binding factor (LEF). The latter is essential for preservation of hematopoietic stem cell functions and thymocyte maturation [10], [11], [12], [13]. However, importance of the Wnt pathway in mature T cells must be better defined. Recent studies in mice demonstrated a link between the development of CD8^+^ T_CM_ cells and activation of the Wnt pathway through accumulation of β-catenin and TCF in T_CM_ but not in T_EM_ [14], [15]. Abolition of TCF expression did not impair the primary response to infections but impeded protection against further infections from the same pathogen. TCF was proven to be critical for secondary expansion of virus-specific CD8^+^ T cells and generation of T_CM_ precursors in mice [14].

Although the canonical Wnt pathway in memory CD8^+^ T cell development seems central in animal models, its relevance to human CD8^+^ T cell biology remains elusive. We therefore evaluated the phenotypic and functional impacts of Wnt pathway activation on human CD8^+^ T cells obtained from peripheral blood mononuclear cells (PBMC) and from tumor infiltrating immune cells (TIIC). We demonstrate that activation of the Wnt pathway with TWS119 generates a population of CD8^+^ T cells showing a young/memory phenotype similar to the T_SCM_ previously described in mice. Furthermore, we report that activation of the Wnt pathway in human CD8^+^ T cells activates mechanisms that could improve the persistence of these cells by an overexpression of Bcl-2 and absence of active caspase-3.

## Materials and Methods

### Normal Donors and Patients

Normal donors were recruited at the Centre de Recherche du Centre Hospitalier de l’Université de Montréal (CRCHUM), Notre-Dame Hospital (Montréal, Canada). Normal donors and lung cancer patients signed an informed consent approved by the Scientific and ethics review committee of the Centre Hospitalier de l’Université de Montréal Research Center (CRCHUM). Blood samples were collected (preceding surgery for patients) and PBMC were isolated following centrifugation with lymphocyte separation medium (Wisent, St-Bruno, Québec, Canada).

Clinical samples from lung tumors were collected shortly after resection (less than 1 hour after surgical resection) and immerged in *Iscove’s modified Dulbecco’s medium* (Invitrogen, Carlsbad, CA). For flow cytometry analysis (FACS), tumor infiltrating immune cells (TIIC) were isolated by mechanical homogenization using a Medimachine™ (Dako Cytomation, Glostrup, Denmark) followed by centrifugation of the resulting single-cell suspension with lymphocyte separation medium (Wisent) [16]. For cell culture, TIIC were isolated by enzymatic digestion as previously described, followed by centrifugation with lymphocyte separation medium [17]. Briefly, tumor samples were sliced in to 1 mm^2^ fragments and digested overnight at room temperature in 10 mL of *RPMI 1640* (Wisent) with type IV collagenase, hyaluronidase, DNase (Sigma-Aldrich, Oakville, Ontario, Canada), 100 U/mL of penicillin/streptomycin and 10 µg/mL of gentamicin (Wisent). Digestion was done on a rotating plate at 50 rpm. Recovered TIIC were resuspended in complete *Iscove’s modified Dulbecco’s medium* supplemented with 7,5% of AB human serum (Gemini Bio-Products, Calabasas, CA), 2 mM L-glutamine, 100 U/mL of penicillin/streptomycin and 10 µg/mL of gentamicin (Iscove), for 18 to 20 h at 37°C with 5% CO_2_ to allow re-expression of faded markers caused by collagen treatment.

### Culture of CD8^+^ T Cells from Normal Donor PBMC or from TIIC

CD8^+^ T cells from PBMC were prepared with *EasySep Negative Selection Human CD8^+^ T Cell Enrichment Kit* (STEMCELL Technologies Inc., Vancouver, BC, Canada) as recommended by the manufacturer. CD8^+^ T cells from TIIC were prepared with *EasySep Positive Selection Human CD8^+^ T Cell Enrichment Kit* (STEMCELL Technologies Inc.) to eliminate cancer cells (>98% purity).

Purified CD8^+^ T cells from either PBMC or from TIIC were cultured in 96-well plates coated with an antibody specific to CD3 (*OKT3*, eBioscience, San Diego, CA; 1 µg/mL for at least 6 h at 4°C or 5 µg/mL for 2 h at 37°C). CD8^+^ T cells were divided into two groups which both received 300 U/mL of IL-2 (Peprotech, Rocky Hill, NJ) on day 0. One group also received a concentration of 5 µM of TWS119 (Calbiochem, Merck KGaA, Darmstadt, Germany), the other group received an equivalent volume of vehicle alone (DMSO). Additional groups were added for dose-response experiments with concentrations ranging from 0.5 to 2.5 µM of TWS119. Cells were cultured for five days in Iscove at 37°C with 5% CO_2_. Complete Iscove’s medium and 600 U/mL of IL-2 were added on day three of culture. After 5 days, CD8^+^ T cells were harvested for either cell surface staining with FACS antibodies or overnight stimulation for cytokine release assays (as below).

### Flow Cytometry

These antibodies were used for cell staining: CD8-Pacific Blue (PB), CD3-Alexa Fluor 700, CD127-Phycoerythrin (PE), CD45RA-Phycoerythrin Cy7 (PECy7), CD62L-Phycoerythrin Cy5 (PECy5) (all from BD Biosciences, Mississauga, ON, Canada) and CD133-APC (eBioscience). Matched isotype controls were used for each antibody to define background levels. Dead cells were excluded from analysis with the *Live/Dead Fixable Dead Cell* kit (Invitrogen). Cell surface staining was performed as described previously [18]. Sample acquisition was performed on a BD Biosciences LSRII instrument and analysis was done using *FlowJo software* (Tree Star). Mean fluorescence intensity (MFI) was calculated on positively stained cells.

### Cytokine Release Assays

In the case of multiplex cytokine detection assays, cells were stimulated for 20 h (37°C, 5% CO_2_) in 96-well plates coated with anti-CD3 (OKT3) and 1 µg/mL of soluble anti-CD28 (BD Biosciences). Supernatants were harvested for cytokine secretion assays by Cytometric Bead Array (CBA, BD Biosciences), or enzyme-linked immunosorbent assay (ELISA). CBA was performed according to the instruction manual of BD Cytometric Bead Array Human Soluble Protein Master Buffer Kit, for these secreted factors: IL-1ß, -2, -4, -5, -6, -7, -9, -10, -12p70, -13, -17a, -21, IFN-γ, GM-CSF, LT-α, TNF, TNF-RI, TNF-RII, CD40L, FasL (CD178), CCL3 (MIP-1α), CCL4 (MIP-1β), CCL5 (RANTES), CXCL8 (IL-8), CXCL9 (MIG), CXCL10 (IP-10) and VEGF. Sample acquisition was performed on a BD Biosciences LSR Fortessa instrument and analysis was done with the FCAP Array software (BD Biosciences). For the human IFN-γ ELISA, 96-well plates (MaxiSorp by Nalge Nunc International, Rochester, NY) were coated overnight with 0.4 µg/mL of anti-IFN-γ antibody (Pierce Biotechnology, Rockford, IL) at 4°C. Plates were washed once with PBS/0.5% tween and blocked for 30 min with PBS with 5% FBS. Plates were washed four times and incubated for 90 min with 0.2 µg/mL of secondary biotinylated anti-IFN-γ antibody (Pierce Biotechnology) and culture supernatants. Plates were washed four times and incubated for 35 min with 0.3 µg/mL of Poly HRP20-streptavidin (Fitzgerald Industries International Inc., Concord, MA). After four washes, plates were revealed with TMB substrate (Neogen, Lexington, KY) and reaction was stopped using 2 N H_2_SO_4_. MIP-1β ELISA was performed according to the manufacturer’s instructions (R&D Systems, Minneapolis, MN). Cytokine secretion was considered positive and specific when values were above 50 pg/mL and double the value of the negative control.

### Western Blotting

Freshly isolated CD8^+^ T cells from healthy donors’ PBMC were stimulated in a 96-well plate pre-coated with anti-CD3 (1 µg/mL) and 1 µg/mL of soluble anti-CD28, with or without TWS119 (5 µM) and harvested after 6 h of incubation (37°C, 5% CO_2_) for β-catenin expression analysis. For Bcl-2 and activated caspase-3 assessment, CD8^+^ T cells were activated for five days as described earlier, with or without 5 µM of TWS119. Following protein extract preparation and quantification as previously described [19], 15–20 µg protein were resolved on 7.5% SDS-PAGE for β-catenin and 15% for Bcl-2 and cleaved caspase-3, and transferred to polyvinylidene difluoride membranes. Membranes were incubated with specific antibodies for β-catenin (mouse anti-β-catenin [IgG1] BD Biosciences, 1/1 000), for Bcl-2 (mouse anti-Bcl-2 [IgG1] Santa Cruz Biotechnology Inc, Santa Cruz, CA, 1/10 000) or for caspase-3 (rabbit anti-caspase-3 [IgG] Santa Cruz Biotechnology Inc, 1/5 000) and for β-actin (mouse anti-β-actin [IgG1] Abcam, 1/75 000). Antibodies were incubated with peroxidase-conjugated goat anti-mouse secondary antibody (Santa Cruz Biotechnology Inc, 1/10 000 [for β-catenin, Bcl-2 and caspase-3] and 1/75 000 [for β-actin]). A peroxidase-conjugated donkey anti-rabbit secondary antibody (Santa Cruz Biotechnology Inc) was used at 1/10 000 for cleaved caspase-3. Proteins were detected using Amersham ECL™ Western Blotting Detection Reagent (GE Healthcare, Buckinghamshire, UK). For Bcl-2, signal intensity was determined by densitometry using Quantity One software (Bio-Rad, Hercules, CA). Basic signal was corrected for background for each band and Bcl-2 densitometric values normalized to β-actin.

### Statistics

In CBA assays, experimental data are reported as mean ± SEM. Statistical analyses were performed by the nonparametric Wilcoxon matched-pairs signed rank test to compare two groups. Statistical analyses were made using Prism 5.0 (GraphPad Software Inc.). P values lower than 0.05 were considered significant.

## Results

### Activation of the Wnt Pathway Leads to the Differentiation of Mature Peripheral Human CD8^+^ T Cells into a Shared Naive, Memory and Stem Cell Phenotype

**Figure 1:**
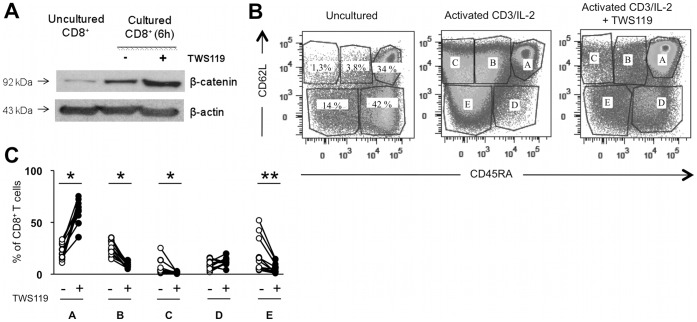
Activation of human CD8^+^ T cells via the Wnt pathway generates cells with a naive phenotype. CD8^+^ T cells from peripheral blood of healthy donors were either untreated or polyclonally activated in the presence of TWS119 (Wnt pathway activator) prior to their analysis. (**A**) Evaluation of β-catenin protein expression by Western blot analysis from fresh CD8^+^ T cells or CD8^+^ T cells activated for 6 h in the presence of anti-CD3 and IL-2, with or without TWS119. β-actin protein level was used as a loading control. (**B**) Representative dot plot analysis of CD45RA and CD62L surface expression on CD8^+^ T cells either fresh (left) or cultured 5 days with anti-CD3 and IL-2, with (middle) or without (right) TWS119. Sub-populations were defined according to isotype controls and population density. (**C**) Percentage of CD8^+^ T cells in each CD45RA/CD62L populations defined in panel B, for 11 healthy donors. * *P* = 0.001; ** *P* = 0.002.

The Wnt pathway is central in multiple differentiation processes and is activated by the pharmacological compound TWS119, which binds to and inhibits GSK-3β [20]. In mice, Gattinoni *et al.* reported that activation of the Wnt pathway with TWS119 in mature anti-gp100 CD8^+^ T cells generated T_SCM_ cells, with a phenotype shared by T_N_, T_CM_ and stem cells [7]. However, Muralidharan *et al*. reported that activation of the Wnt pathway in mature human CD8^+^ T cells blocked naive to effector transition [9] without emphasizing the young memory phenotype reported by Gattinoni *et al.* [7], [8], [21]. We thus isolated CD8^+^ T cells from circulating blood of healthy donors and activated them with anti-CD3 and IL-2 to mimic TCR activation, in the presence or absence of TWS119 for five days. As expected, treatment with TWS119 stabilized β-catenin in human activated CD8^+^ T cells (Figure 1A). Following activation, phenotypic profile of total CD8^+^ T cells was established using flow cytometry analysis (FACS). At the time of this current study, T_SCM_ have yet only been reported in mice [6], [7], therefore we selected their human ortholog CD45RA and CD62L to evaluate the impact of Wnt pathway activation on shared T_N_ and T_CM_ human CD8^+^ T cell phenotypes. As shown in Figure 1B, activation of CD8^+^ T cells combined with TWS119 treatment (Figure 1B, right dot plot) resulted in distinct populations when compared with activated CD8^+^ T cells without TWS119 (Figure 1B, center dot plot) with a defined CD45RA^hi^/CD62L^+^ population (gate A) of CD8^+^ T cells. This three-fold increase in population A was observed in cells from all healthy donors treated with TWS119 (Figure 1C). For the other populations, T cell activation with TWS119 resulted in marginal differences in the percentage of cells (population D) or in more (population E) or less (populations B and C) of a decrease.

**Figure 2:**
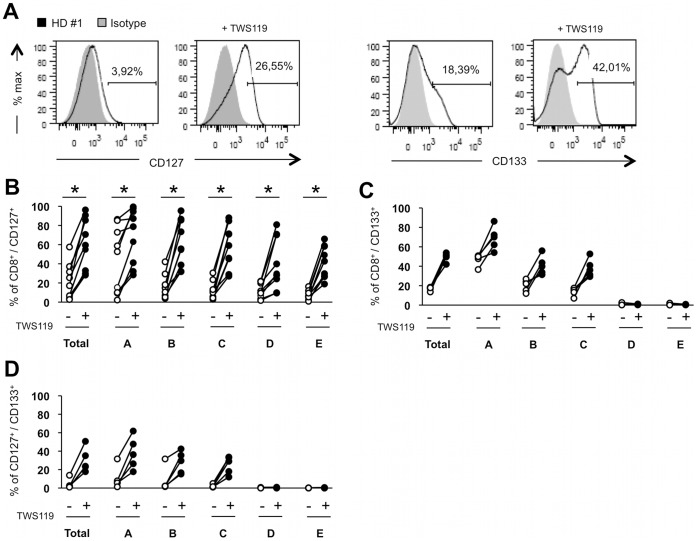
Activation of human CD8^+^ T cells in the presence of TWS119 favors a young/memory phenotype. CD127 and CD133 expression in CD8^+^ T cell sub-populations defined by expression of CD45RA and CD62L (see Figure 1A) following treatment with TWS119. (**A**) Representative histograms of total CD8^+^ T cells expressing CD127 or CD133 after a 5-day anti-CD3/IL-2 activation with or without TWS119. (**B–C**) Percentage of CD8^+^ T cells expressing, respectively, CD127 (**B**) and CD133 (**C**) in total CD8^+^ T cell population (Total), and in each CD45RA/CD62L-defined population from Figure 1A, for 9 healthy donors in B and 5 in C. (**D**) Percentage of CD8^+^ T cells expressing both CD127 and CD133 markers in total CD8^+^ T cell population (total) and in each CD45RA/CD62L-defined population from Figure 1A for 5 healthy donors. * *P*<0.004.

To further characterize the impact of Wnt activation on human CD8^+^ T cell phenotype, expression of the IL-7α receptor (CD127) was evaluated as a marker for persisting and/or memory cells [22]. In addition, expression of the CD133 molecule was also investigated as a putative stem cell marker [23]. CD133 expression has previously been partly linked to Wnt pathway activation in human fetal aorta progenitor cells, but remains unexplored in human T cells [24]. As presented in Figure 2A (left) and 2B (Total population), activation of CD8^+^ T cells for five days combined with TWS119 treatment resulted in both increased percentage and intensity of CD127 (Figure S1, left). Although we observed some variability between healthy donors, expression of CD127 was increased in all defined populations from Figure 1B according to CD45RA and CD62L expression. This increase (Figure 2B) was even observed in population E (CD45RA^−/^CD62L^−^), considered of effector phenotype in which most CD8^+^ T cells do not normally express CD127. This demonstrated an impact of Wnt pathway activation even in differentiated cells. Similar observations were made for CD133 (Figure 2C and Figure S1, right). However, CD133 was absent from the CD62L-negative population (populations D and E in Figure 2C). As illustrated in population A (Figure 2D), a high percentage of CD8^+^ T cells expressing CD45RA^hi^/CD62L^+^/CD127^+^/CD133^+^ is generated when TWS119 is added to cultures. This indicates that by activating the Wnt pathway in human CD8^+^ T cells, it is possible to induce a T_SCM_-like phenotype. Such treatment also increases co-expression of CD127 and CD133 in CD8^+^ T CD45RA^int^/CD62L^+^ and CD45RA^−/^CD62L^+^ cells (respectively populations B and C in Figure 2D).

### TWS119 Dose-response and CD8^+^ T_SCM_ Establishment

**Figure 3:**
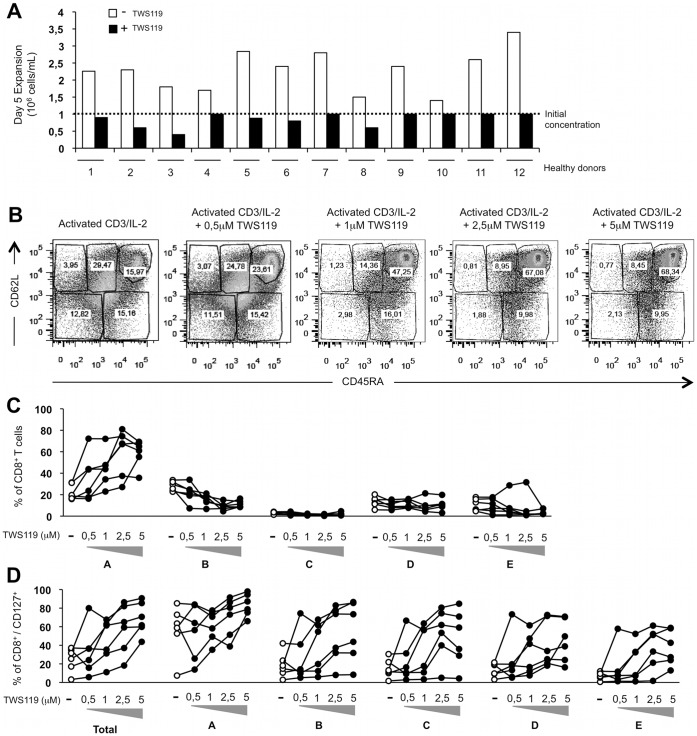
A minimal concentration of 2.5 µM of TWS119 is required to generate T cells with a young/memory phenotype. Expression of CD45RA and CD62L on CD8^+^ T cells from peripheral blood of healthy donors after activation of the Wnt pathway with TWS119 dose-response (0.5 to 5 µM). (**A**) Expansion of viable CD8^+^ T cells activated with anti-CD3/IL-2 after five days of culture with or without 5 µM TWS119, illustrated by final cell concentration. Initial cell concentration was 1×10^6^ cell/mL. (**B**) Representative dot plot analysis for surface expression of CD45RA and CD62L on CD8^+^ T cells activated with anti-CD3/IL-2 with or without increasing concentrations of TWS119. (**C**) Percentage of total CD8^+^ T cells in each CD45RA/CD62L population for 6 healthy donors. (**D**) Percentage of CD8^+^ T cells expressing CD127 in total CD8^+^ T cell population in a TWS119 dose-response, in each population defined by expression of CD45RA and CD62L in Figure 1A (for 6 donors).

We next elected to determine whether the Wnt pathway modifies CD8^+^ T cell expansion. As shown in Figure 3A, high concentrations of TWS119 impair human CD8^+^ T cell expansion, which is also observed in mice [7]. Despite a lower number in the total cell number raised, the absolute number of T_SCM_-like cells may be higher following TWS119 treatment due to the increase in the proportion of these cells. This T_SCM_-like phenotype increase cannot be attributed to death of other T cell types (populations B to E) since the number of dead cells was similarly low in both groups whether treated or not with TWS119, with some donor-specific variability (Figure S2).

To overcome this apparent lack of expansion in the total cell population, peripheral blood human CD8^+^ T cells were activated for five days in a dose-response of TWS119 (0.5 to 5 µM) to find an intermediate concentration allowing T_SCM_ differentiation and exerting minimal impact on cell number. As presented in Figure 3B, distinct CD45RA^hi^/CD62L^+^ (population A as defined in Figure 1B) peaked with 2.5 and 5 µM TWS119. This was observed for most healthy donors (Figure 3C). Donors with a conserved CD45RA^hi^/CD62L^+^ phenotype from 2.5 to 5 µM concentration also showed a lack of expansion potential with 2.5 µM (Figure S3). Considering the variations between healthy donors obtained in our cultures with lower doses of TWS119 and absence of specific toxicity, 5 µM of TWS119 appeared optimal to preserve the T_SCM_-like phenotype acquired following Wnt pathway activation, but cells poorly proliferated at this concentration.

Increased concentration of TWS119 was also linked to an increase of CD127 in total CD8^+^ T cells (Figure 3D and Figure S4 for MFI) but also in all sub-populations defined in Figure 1B (populations A to E, Figure 3D). Again, these data confirmed that 5 µM of TWS119 is optimal to induce the shared naive/memory/stem T_SCM_-like phenotype, in combination with IL-2.

### Impact of Wnt Pathway Activation on Human CD8^+^ T Cell Functions

**Figure 4:**
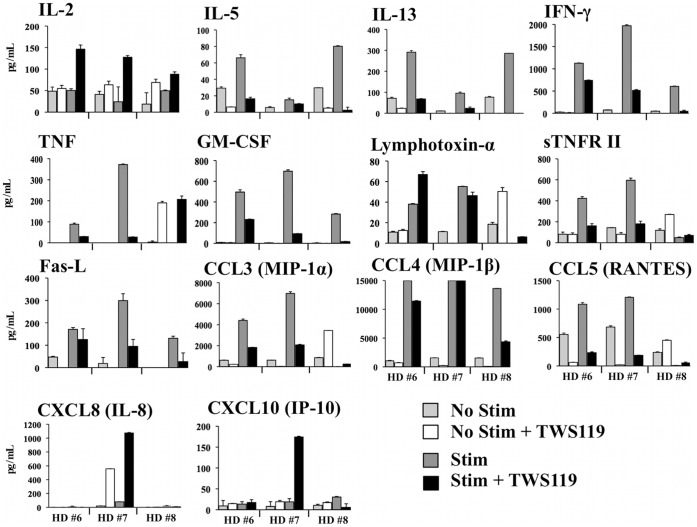
Activation of the Wnt pathway in human CD8^+^ T cells modulates secretion of effector cytokines/chemokines and secreted factors. Cytokine secretion profile of total CD8^+^ T cells from peripheral blood of healthy donors following the Wnt pathway activation with TWS119. Cytokine secretion was measured by CBA with a panel of 27 cytokines in supernatant of total CD8^+^ T cells cultured with anti-CD3/IL-2 and TWS119 and reactivated with anti-CD3/anti-CD28 and TWS119 (data from 3 healthy donors). Only cytokines considered positive for secretion (>50 pg/mL and double or higher the “No Stimulation” value) are presented (negative for IL-1ß, -4, -6, -7, -9, -10, -12p70, -17a, -21, CD40L, TNF-RI, CXCL9 (MIG) and VEGF).

Since the Wnt pathway mediated major changes in the phenotype of human CD8^+^ T cell, we elected to investigate whether this pathway also impacts on CD8^+^ T cell functionality. To fully establish the polyfunctional secretion profile, peripheral blood human CD8^+^ T cells were activated for five days with or without TWS119 and restimulated overnight with agonist anti-CD3 and anti-CD28 antibodies. Supernatants were collected and assayed for the presence of 27 different cytokines/chemokines/immune mediators. As presented in Figure 4, activation of the Wnt pathway in CD8^+^ T cells diminished the secretion of effector cytokines, chemokines and modulators such as IFN-γ, GM-CSF, TNF, sTNFRII, MIP-1 (α and β) and RANTES, but increased (vrai?) IL-2 secretion for the majority of donors. Results for IFN-γ and MIP-1β were confirmed by ELISA assays (data not shown). Activation of CD8^+^ T cells also led to production of Th2 cytokines IL-13 and IL-5, which was noticeably diminished in the presence of TWS119. The shift from a clear effector profile to a less differentiated profile combined with secretion of IL-2 is consistent with the young/memory phenotype generated by the activation of the Wnt pathway in human CD8^+^ T cells.

### Activation of the Wnt Pathway in Human Tumor-infiltrating CD8^+^ T Cells Generates a T_SCM_-like Population

**Figure 5:**
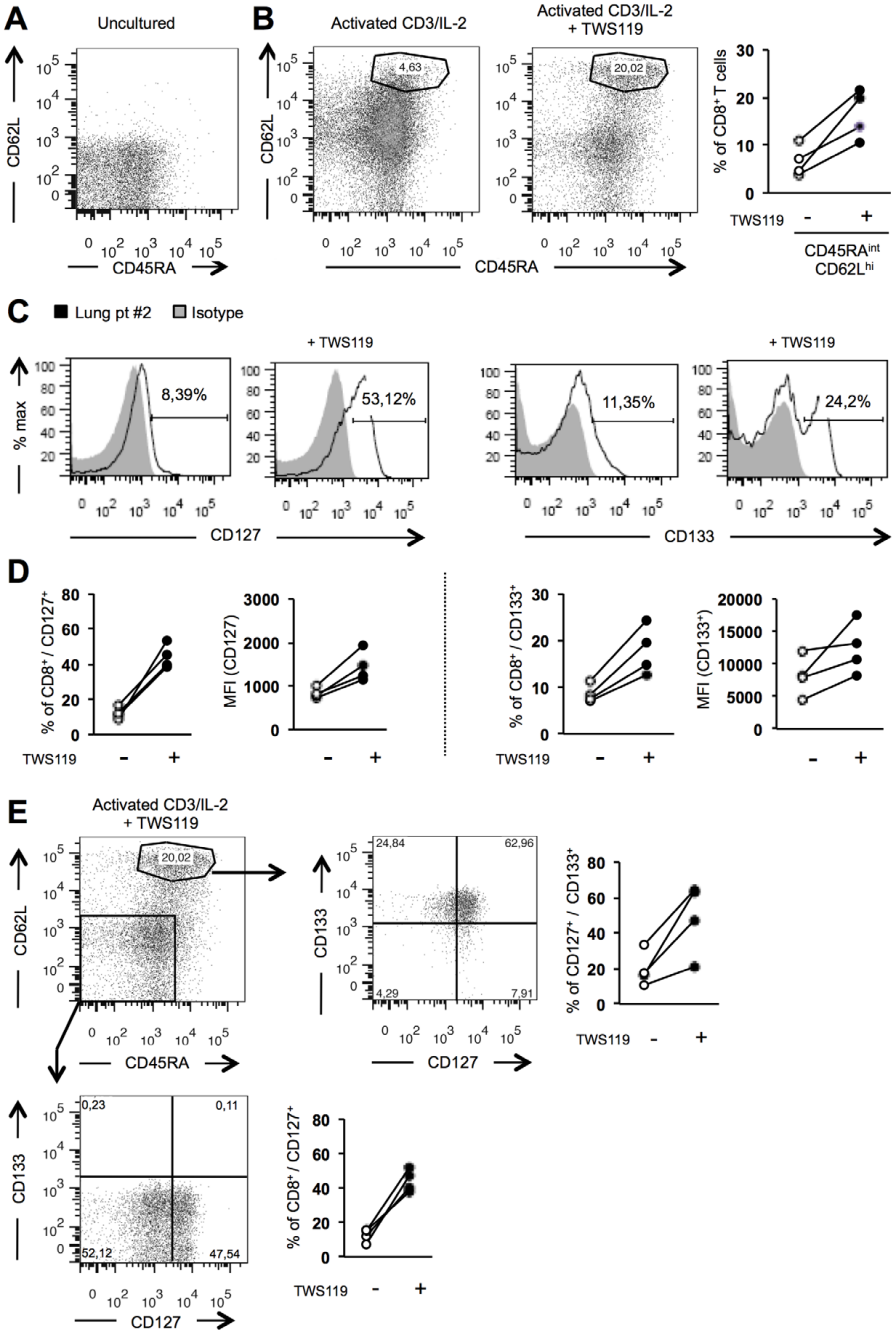
Human CD8^+^ TIL activated via the Wnt pathway acquire a young/memory phenotype. CD8^+^ T cells isolated from lung TIL were cultured with TWS119 and expression of CD45RA, CD62L, CD127 and CD133 was determined by flow cytometry. (**A**) Representative flow cytometry analysis for CD45RA and CD62L expression on CD8^+^ TIL freshly-isolated from lung cancer tumors (representative of 4 tumors). (**B**) Representative analysis of CD45RA/CD62L expression on CD8^+^ TIL following 5 days of anti-CD3/IL-2 activation, with or without TWS119. Compilation graph from 4 patients for the CD45RA^int^/CD62L^+^ defined population (right panel). (**C**) Representative histogram analysis of CD127 (left) or CD133 (right) expression in total CD8^+^ TIL following 5 days of anti-CD3/IL-2, with or without TWS119. (**D**) Compilation graphs for CD127 and CD133 expression (percentage and MFI as indicated) on total activated CD8^+^ TIL. (**E**) Characterization of CD127 and CD133 expression by different CD45RA/CD62L-defined CD8^+^ TIL, following treatment with TWS119. Specifically, expression of CD127 and CD133 on CD45RA^int^/CD62L^+^ and CD45RA^−^/CD62L^−^ CD8^+^ TIL populations is shown, after culture with TWS119.

One of the challenges in the treatment of cancer patients by ACT is to enhance the survival of transferred anti-tumor T cells. Tumor infiltrating T lymphocytes (TIL) have been shown to be more efficient in ACT, but mostly display an activated and further differentiated phenotype (CD45RO^+^, MHC class II^+^) [25] and the majority of these cells are CD45RA^−/^CD62L^−^ (Figure 5A). We thus evaluated whether activation of the Wnt pathway could revert CD8^+^ TIL to a less differentiated phenotype. As shown in Figure 5B, human CD8^+^ TIL prepared from lung cancer patients and activated (anti-CD3/IL-2) in the presence of TWS119 for five days displayed a distinct population of CD45RA^int^/CD62L^+^ cells. This observation was consistent with all four lung cancer patients (Figure 5B, right panel). We also observed variability in TIL viability from patient to patient (Figure S5). Because the viability appeared patient-dependent, we believe that the dose used for TWS119 treatment (5 µM) was not the cause of viability issues. Other factors such as susceptibility of TIL to cell death-activation as a consequence of higher doses of anti-CD3 could also be the cause of cell death observed with some TIL.

Similarly, a greater proportion of CD8^+^ TIL activated in the presence of TWS119 acquired CD127 and CD133 compared to activated cells without the Wnt pathway activator (Figure 5C and D). Also, to a lesser extent, treatment of TIL with TWS119 increased their CD133 expression (Figure 5C right and 5D right). Interestingly, the CD45RA^int^/CD62L^+^ population observed following TWS119 treatment contained a substantial cell population (20–62%) co-expressing CD127 and CD133 (Figure 5E, top panels), which corresponds to a T_SCM_-like phenotype (CD45RA^int^/CD62L^+^/CD127^+^/CD133^+^). Finally, TWS119 failed to upregulate CD133 in CD45RA^−^/CD62L^−^ cells but still caused an increase in CD127 (Figure 5E, bottom panels).

In summary, activation of CD8^+^ lung TIL in the presence of TWS119 gives rise to a distinct cell population with a T_SCM_ phenotype (CD45RA^int^/CD62L^+^/CD127^+^/CD133^+^) when compared to basic CD3/IL-2 activation. Activation of the Wnt pathway in TIL also resulted in higher expression of CD127 in CD45RA^−/^CD62L^−^ CD8^+^ TIL, which may confer a higher capacity to persist following transfer in patients.

### The Wnt Pathway Changes Human Tumor-infiltrating CD8^+^ T Cell Functionality

**Figure 6:**
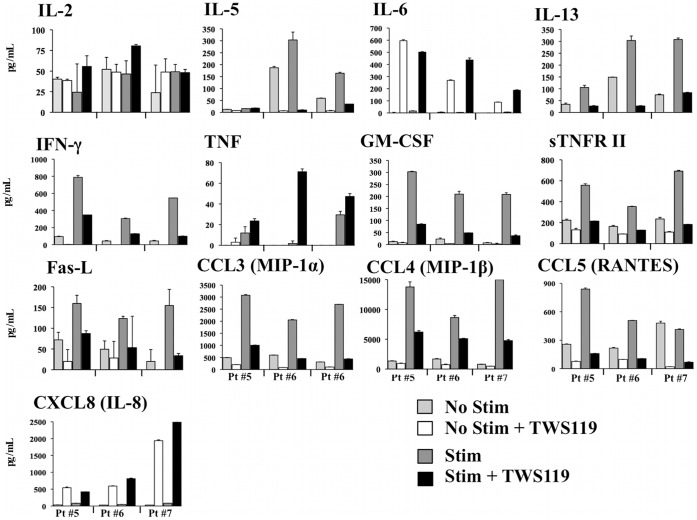
Activation of the Wnt pathway in human CD8^+^ TIL from lung cancer tumors with TWS119 diminishes secretion of effector cytokines. Cytokine secretion profile of CD8^+^ TIL from lung cancer tumors after activation of the Wnt pathway with TWS119. Cytokine secretion was measured by CBA with a panel of 27 cytokines in supernatant of total CD8^+^ TIL cultured with anti-CD3/IL-2 and TWS119 and reactivated overnight with anti-CD3/anti-CD28 and TWS119 (for 3 lung cancer tumors). Only cytokines considered positive for secretion (>50 pg/mL and double or higher the “No Stimulation” value) are presented (negative for IL-1ß, -4, -7, -9, -10, -12p70, -17a, -21, CD40L, LT-α, TNF-RI, CXCL9 (MIG), CXCL10 (IP-10) and VEGF).

Global secreted factors were analyzed from Wnt-activated human tumor-infiltrating CD8^+^ T cells. CD8^+^ lung TIL were activated with or without TWS119 for five days and restimulated overnight with agonist anti-CD3 and anti-CD28. Supernatants were then collected to evaluate 27 different cytokines/chemokines/secreted factors. As presented in Figure 6, activation of the Wnt pathway in CD8^+^ T cells diminished the secretion of some effector cytokines such as IFN-γ, GM-CSF, MIP-1 (α and β) and RANTES when compared with anti-CD3 activation without TWS119. Results for IFN-γ were validated by ELISA (data not shown). Some cytokines showed a distinct response to TWS119; TNF production was higher when anti-CD3 was combined to TWS119, and TWS119 alone increased IL-6 and IL-8 secretion with or without anti-CD3 stimulation. Activation of CD8^+^ lung TIL also led to production of IL-13 and IL-5, which were noticeably diminished in presence of TWS119 as observed with peripheral CD8^+^ T cells (Figure 4). Collectively, Wnt/ß-catenin activation led to a decreased production of pro-inflammatory cytokines which is consistent with less differentiated T cell functions, but also increased the production of IL-6, IL-8, and TNF.

### Wnt/ß-catenin Activation Increases Bcl-2 and Inhibits Caspase-3 Cleavage

**Figure 7:**
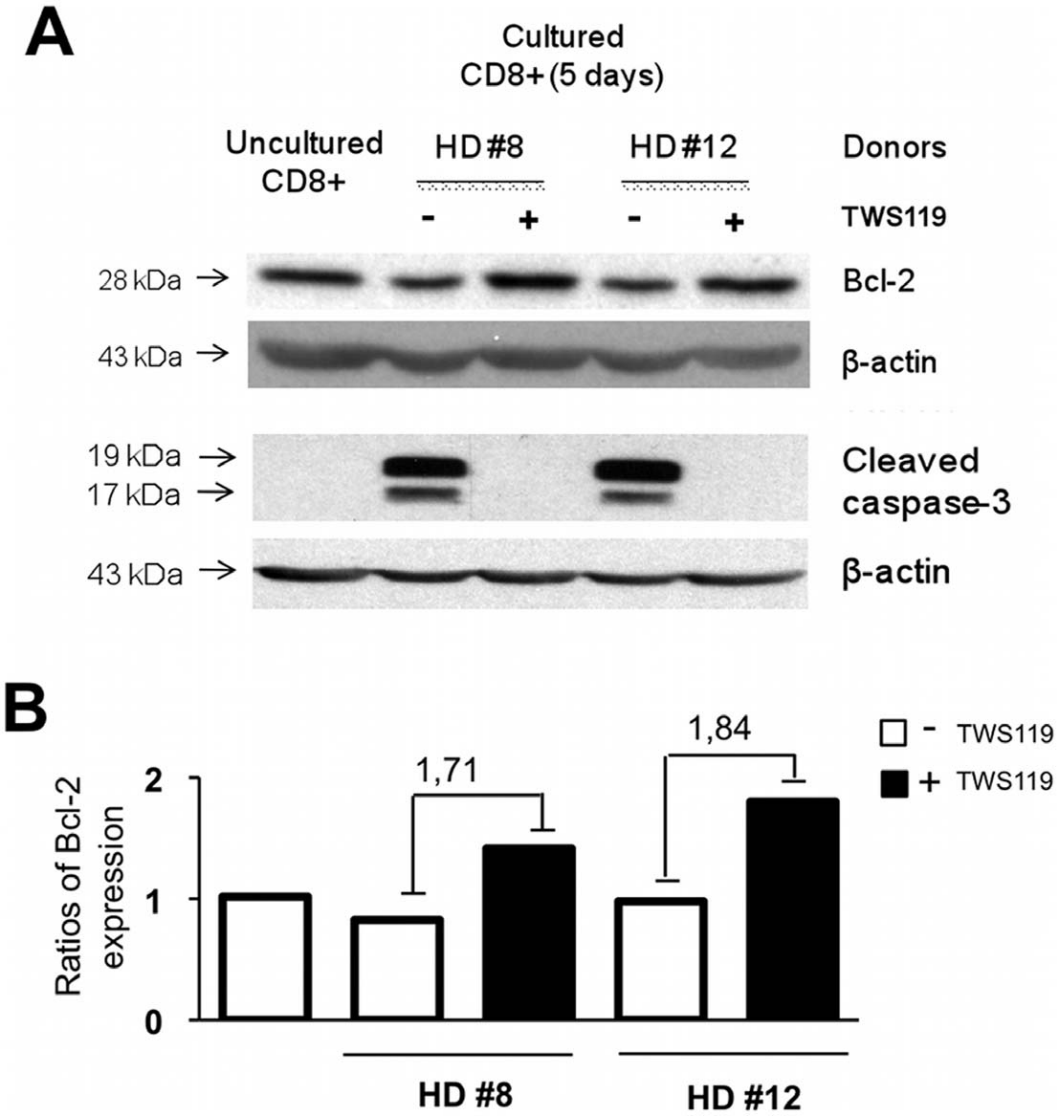
Treatment of human CD8^+^ T cells with TWS119 protects against apoptosis. Molecular analysis of anti- and pro-apoptosis actors in CD8^+^ T cells following activation of the canonical Wnt pathway. (**A**) Densitometric profile of Western blot analysis of the Bcl-2 protein. Data are presented as ratios of Bcl-2 protein expression (normalized with β-actin) and fold changes between CD8^+^ T cells cultured with and without TWS119 for 2 healthy donors (representative of 5). (**B**) Western blot analysis of the cleaved form of caspase-3 (2 fragments) in total CD8^+^ T cells following 5 day anti-CD3/IL-2 activation with or without TWS119 for 2 healthy donors (representative of 5).

Expression of CD127 is presumed to enhance cell survival [22]. Accordingly, treatment of human CD8^+^ T cells with TWS119 not only increased CD127 expression (Figure 2B and 5D) but also enhanced levels of anti-apoptotic protein Bcl-2 expression (Figure 7A and B), a protein protecting from programmed cell death or apoptosis. Furthermore, absence of cleaved caspase-3 (active form) in CD8^+^ T cells treated with TWS119 (Figure 7A) emphasized the idea that activation of the Wnt pathway protected these cells from apoptosis. The nearly 2-fold increase in Bcl-2 combined with the absence of cleaved caspase-3 strongly suggests an enhancement in survival potential mediated by Wnt pathway activation in CD8^+^ T cells. With increased Bcl-2 and absence of detectable caspase-3 activity following TWS119 treatment, cells demonstrated enhanced survival.

## Discussion

Perspectives for cancer treatment by ACT seem very promising but there are still obstacles to overcome, one of them being the persistence of cells following transfer. Long-term persistence is desirable to establish passive transfer of memory response, and consequently avoid cancer recurrence. In an attempt to provide solutions to this problem and to better understand the complex biology of T lymphocyte differentiation, we studied the impact of the canonical Wnt/ß-catenin pathway activation on human CD8^+^ T cells. This pathway has been shown to be important in the establishment of central memory T cells and for prolonged persistence of mouse CD8^+^ T cells following transfer in the B16 mouse model and peptide vaccination [7], [14], [15]. We report here that activation of the Wnt/ß-catenin pathway by treatment with TWS119 generates cells with a shared phenotype between T_N_, T_CM_ and stem cells, similar to T_SCM_ previously described in mice, in human CD8^+^ T cells from peripheral blood, and for the first time from TIL. As for the effect of TWS119 on peripheral CD8^+^ T cells, this report differs in part from the study by Muralidharan *et al.*, which focuses on the naive to effector transition. Also, culture conditions differ in our study; they used lower concentrations of TWS119 without exogenous cytokines for a 7-day culture period [9]. Our cultures included cytokines such as IL-2, which should provide T cell survival signals. Also, our study focused on the impact of Wnt pathway activation in the generation of human T_SCM_-like phenotype, as opposed to the previous study [9]. We also demonstrated the impact of Wnt/ß-catenin pathway activation in cells with a more differentiated profile found in peripheral blood and TIL. These T_SCM_-like cells expressed elevated levels of molecules such as the IL-7α receptor and Bcl-2, in the absence of active caspase-3, supporting the notion that they bear enhanced survival properties.

A recent study reported rare T_SCM_ CD8^+^ and CD4^+^ T cell populations (2–3% of all circulating T lymphocytes) naturally arising in humans [8]. However, Gattinoni et al. established no link between the Wnt/ß-catenin pathway and this naturally-arising T_SCM_ phenotype in humans, and did not report stem cell markers. In our study, the hematopoietic stem cell maker CD133 was clearly expressed on a substantial fraction of T_SCM_ cells raised with TWS119 treatment, with an apparent link with expression of CD62L, which we did not further characterize. Also, it was not clear what was the proliferative capacity of human T_SCM_. Whether putative naturally-arising T_SCM_ described in Gattinoni *et al.* and the Wnt/ß-catenin-activated CD8^+^ T cells we describe are the same population remains to be confirmed in humans, and considering their published microarray data, it is unlikely since Wnt-pathway targeted genes remained unchanged. However, human cells with these attributes have an improved persistence capacity when adoptively-transferred into a xenogeneic mesothelioma mouse tumor model [8].

TWS119 capacity to activate the canonical Wnt/ß-catenin pathway was already recognized in mice [7], [20], but only one study reported its effect on human cells [26]. Specifically, TWS119 is an inhibitor of GSK-3β, which itself inhibits the canonical Wnt pathway. Here, we have shown that treatment of human CD8^+^ T cells with TWS119 induced an accumulation of β-catenin (Figure 1A), as recently reported by Muralidharan *et al.* [9] We also observed a slight increase in ß-catenin following anti-CD3/IL-2 activation, as previously reported by *Lovatt and Bijmaker* who demonstrated in human mature T cells that stimulation of the TCR (anti-CD3 and anti-CD28) leads to β-catenin stabilization early after activation [27]. This concurs with reports of GSK-3 inactivation upon TCR stimulation [28]. Interestingly, GSK-3β is also involved in other pathways, including cellular metabolism, cell cycle progression, neuroprotection and the mTOR pathway [29], [30]. It is therefore plausible that TWS119 affects other pathways in addition to stabilizing β-catenin, which could explain the T_SCM_ phenotype. However, it is unlikely that TWS119 engages the mTOR pathway after GSK-3β inhibition, since mTOR engagement classically leads to proliferation, which does not happen when human CD8^+^ T cells are treated with TWS119, considering they fail to expand (Figure 3A). Moreover, activation of the Wnt pathway seems to dominate over activation of the mTOR pathway [31]. Furthermore, introduction of stable ß-catenin in peripheral mouse primary T cells inhibited proliferation and cytokine secretion after TCR stimulation and blunted effector cell differentiation [32]. However, the same groups reported that the sole stabilization and expression of ß-catenin may not be sufficient to lead to a T_SCM_ phenotype; it is therefore possible that other pathways involving GSK-3ß may be essential [33]. Consequently, as the sole involvement of Wnt/ß-catenin pathway for T_SCM_ differentiation has been a matter of debate (Driessens, Correspondence to the editor, Nat Med, 2010 and Reply from Gattinoni Nat Med, 2010) [33], importance of this pathway requires further investigation, even though it has been replicated in other mouse models [14], [15]. Still, although in addition to ß-catenin other pathways may be engaged, GSK-3ß inhibition by TWS119 resulted in a T_SCM_ phenotype in human T CD8^+^ cells.

We also observed an increase in IL-7α receptor expression in the total population of CD8^+^ T cells, independently of their source (Figure 2B, 5D and Figure S1A). Cells expressing high CD127 levels are known to have the highest capacity to become long persisting memory cells. During an efficient immune response, they represent a small subset during the peak of response but persist after contraction [22], [34]. TWS119 differentiates CD8^+^ T_CM_-like cells (CD62L^+^/CD127^+^), but also increases CD127 expression in populations that would be considered differentiated effectors with limited life expectancy, based on absence of CD62L and CD45RA expression [35]. CD133 expression represents a hallmark marker of hematopoietic stem cells, and is also expressed as an early myogenic marker [36], but has never been reported in mature T lymphocytes. Thus, as CD133 may be a unique T_SCM_ marker, further characterization of this population is required.

The CD8^+^ T_SCM_-like cells we generated from both PBMC and TIL secreted a wide array of cytokines, a concept referred to as polyfunctionality. It has recently been demonstrated in mouse models, that while fully differentiated highly proliferating effector CD8^+^ T cells are mostly IFN-γ^+^/TNF^−^ following antigen recognition, less differentiated cells are more likely to be IL-2^+^/TNF^+^ with progressive acquisition of IFN-γ [37]. Although our study was performed in humans, there are similarities regarding the partial loss of IFN-γ production combined to gain of IL-2 production following activation of the Wnt/ß-catenin pathway in peripheral human CD8^+^ T cells, also observed in human CD8^+^ TIL. Similar observations were made previously [9] on decreased IFN-γ secretion by T cells treated with TWS119, with a weak decrease in TNF production, although they observed appreciable amounts of IL-2 independently of Wnt pathway activation. Conversely, we demonstrated that Wnt activation in human CD8^+^ TIL increased TNF production compared to non-TWS119 treated controls. Comparison remains suboptimal since TIL are already activated and display a different biology compared to peripheral T cells.

Although we cannot exclude that inhibition of GSK-3 could lead to activation of the nuclear factor of activated T cell (NF-AT) pathway, thus leading to secretion of IL-2 by CD8^+^ T cells, activation of this pathway in unlikely because it would also result in T cell proliferation, which does not concur with our results [28], [38]. Globally, when looking at the diversity of secreted products, we observed that treatment with TWS119 preserves cells from acquisition of a fully effector phenotype by a decrease in effector cytokines, which has also been reported in mice and recently in humans [7], [9]. Interestingly, even with this decrease of cytokines and secreted factors, such a less differentiated phenotype has proven to be more effective to eradicate large tumors by ACT treatment [21], which has been partly repeated with naturally-arising T_SCM_ (Gattinoni). Thus, activation of the Wnt pathway confers expression of a lymphoid homing receptor that is known to represent a key element for effective tumor response, but also expression of Bcl-2 and loss of active caspase-3, both favoring persistence and protection from apoptosis to human CD8^+^ T cells from the periphery and infiltrating tumors.

In conclusion, our study is the first to explore the activation of the Wnt/ß-catenin pathway in human CD8^+^ T cells from tumor infiltrate, and provides a basis to extend research towards antigen-specific models. These include T cells genetically-engineered to express T cell receptors or chimeric antigen receptors (CAR) which can be exploited with human CD8^+^ T cells and could provide new insights on the impact of Wnt/ß-catenin pathway activation and extended persistence of these cells following ACT. As T_SCM_ may also present different subtypes (based on CD133 expression and cytokine production, for example), more studies are needed to unravel the precise function and potential use of these cells.

## Supporting Information

Figure S1 **Mean Fluorescence Intensity (MFI) of both CD127 and CD133 evaluated on total CD8^+^ T cells activated with anti-CD3 and IL-2 for five days with or without TWS119.** MFI of CD127 (A) was evaluated on CD8^+^ T cells of 11 healthy donors (HD) and CD133 (B) on 5 HD.
(TIFF)

Figure S2 **Percentage of live CD8^+^ T cells activated with anti-CD3/IL-2 following five days of culture with or without TWS119.** Cell viability was evaluated by flow cytometry by gating on cells that excluded the viability dye (LIVE/DEAD® Fixable Aqua Dead Cell Stain Kit, Invitrogen).
(TIFF)

Figure S3 **Proliferation of CD8^+^ T cells activated with anti-CD3/IL-2 following five days of culture with a TWS119 dose-response (0.5 μM to 5 μM), illustrated by final cell concentration.** Cell counts were performed manually and viability evaluated by exclusion of trypan blue.
(TIFF)

Figure S4 **Evaluation of CD127 MFI of total CD8^+^ T cells activated with anti-CD3 and IL-2 for five days with a TWS119 dose-response (0.5 to 5 μM).** Eleven HD were evaluated for CD127 and 5 HD for CD133.
(TIFF)

Figure S5 **Percentage of live CD8^+^ TIL from lung cancer activated with anti-CD3/IL-2 after five days of culture with or without TWS119.** Cell viability was evaluated by flow cytometry by gating on cells that excluded the viability dye (LIVE/DEAD® Fixable Aqua Dead Cell Stain Kit, Invitrogen).
(TIFF)
