# Supplementary figures and images for: Stimulation of Wnt/ß-Catenin Pathway in Human CD8+ T Lymphocytes from Blood and Lung Tumors Leads to a Shared Young/Memory Phenotype
Source: PLoS One. 2012 Jul 30;7(7):e41074. doi: 10.1371/journal.pone.0041074 (PMC3408435; doi:10.1371/journal.pone.0041074)

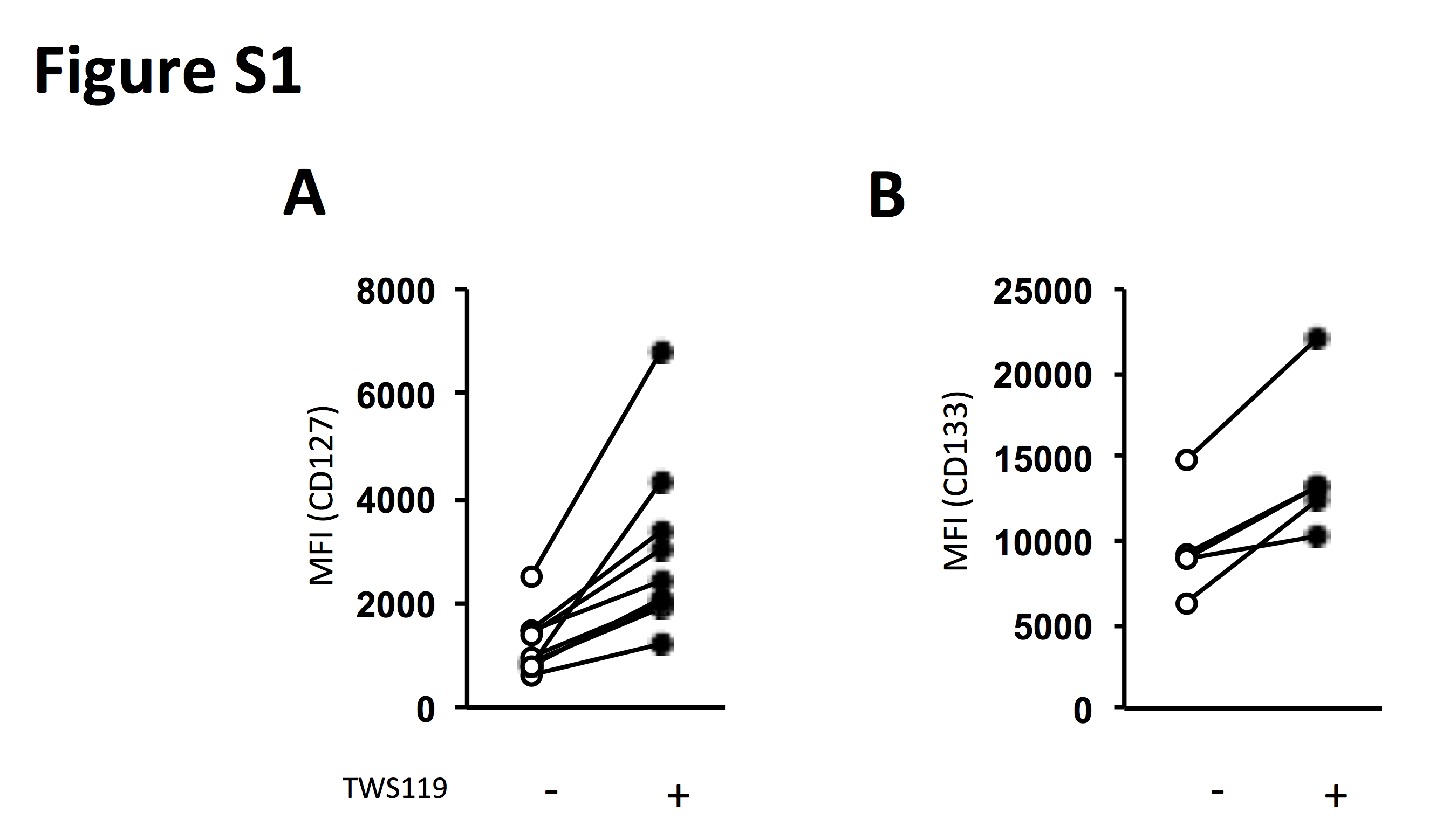

Supplement: Figure S1 — Mean Fluorescence Intensity (MFI) of both CD127 and CD133 evaluated on total CD8+ T cells activated with anti-CD3 and IL-2 for five days with or without TWS119. MFI of CD127 (A) was evaluated on CD8+ T cells of 11 healthy donors (HD) and CD133 (B) on 5 HD. (TIFF) [file pone.0041074.s001.tiff]

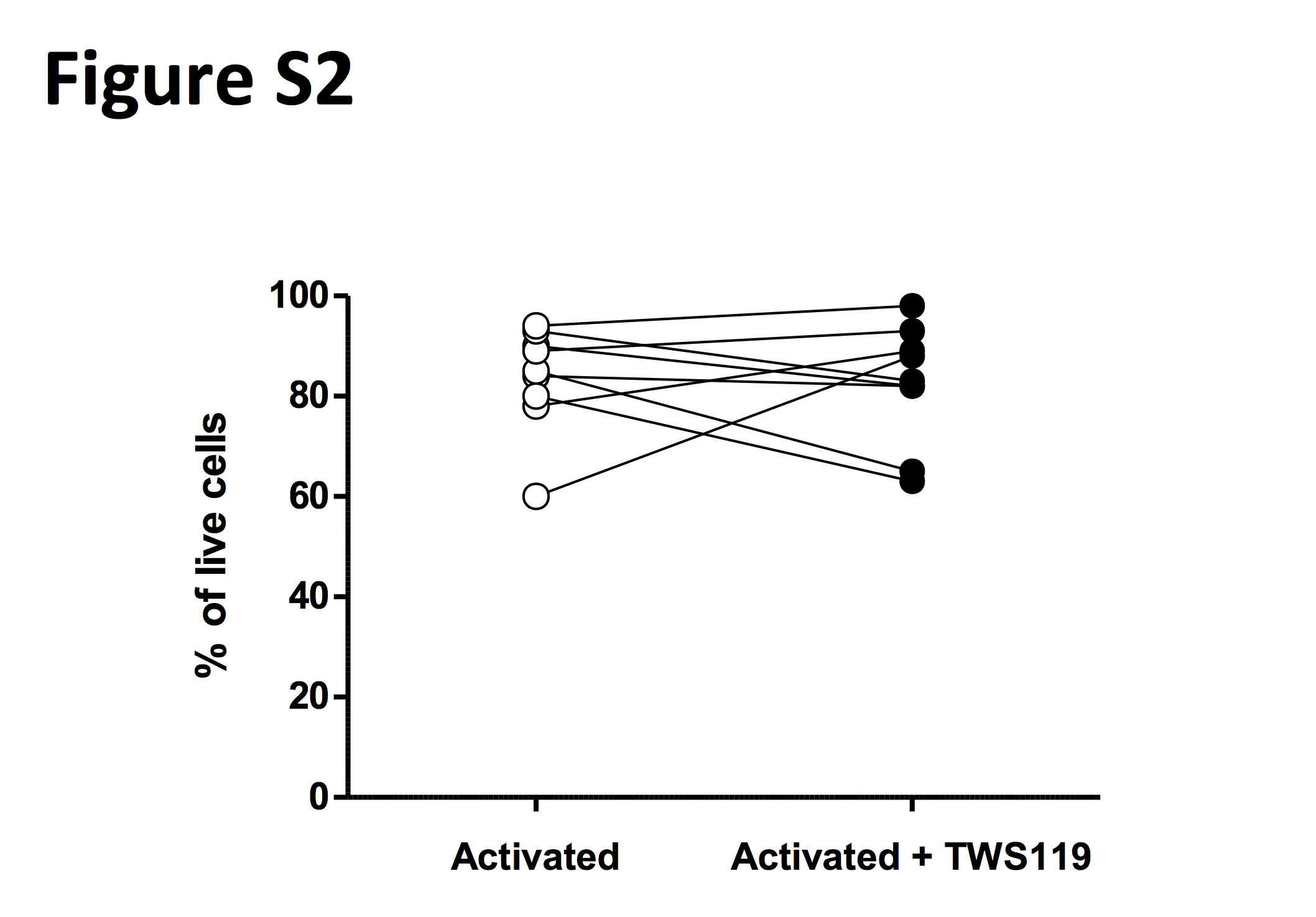

Supplement: Figure S2 — Percentage of live CD8+ T cells activated with anti-CD3/IL-2 following five days of culture with or without TWS119. Cell viability was evaluated by flow cytometry by gating on cells that excluded the viability dye (LIVE/DEAD® Fixable Aqua Dead Cell Stain Kit, Invitrogen). (TIFF) [file pone.0041074.s002.tiff]

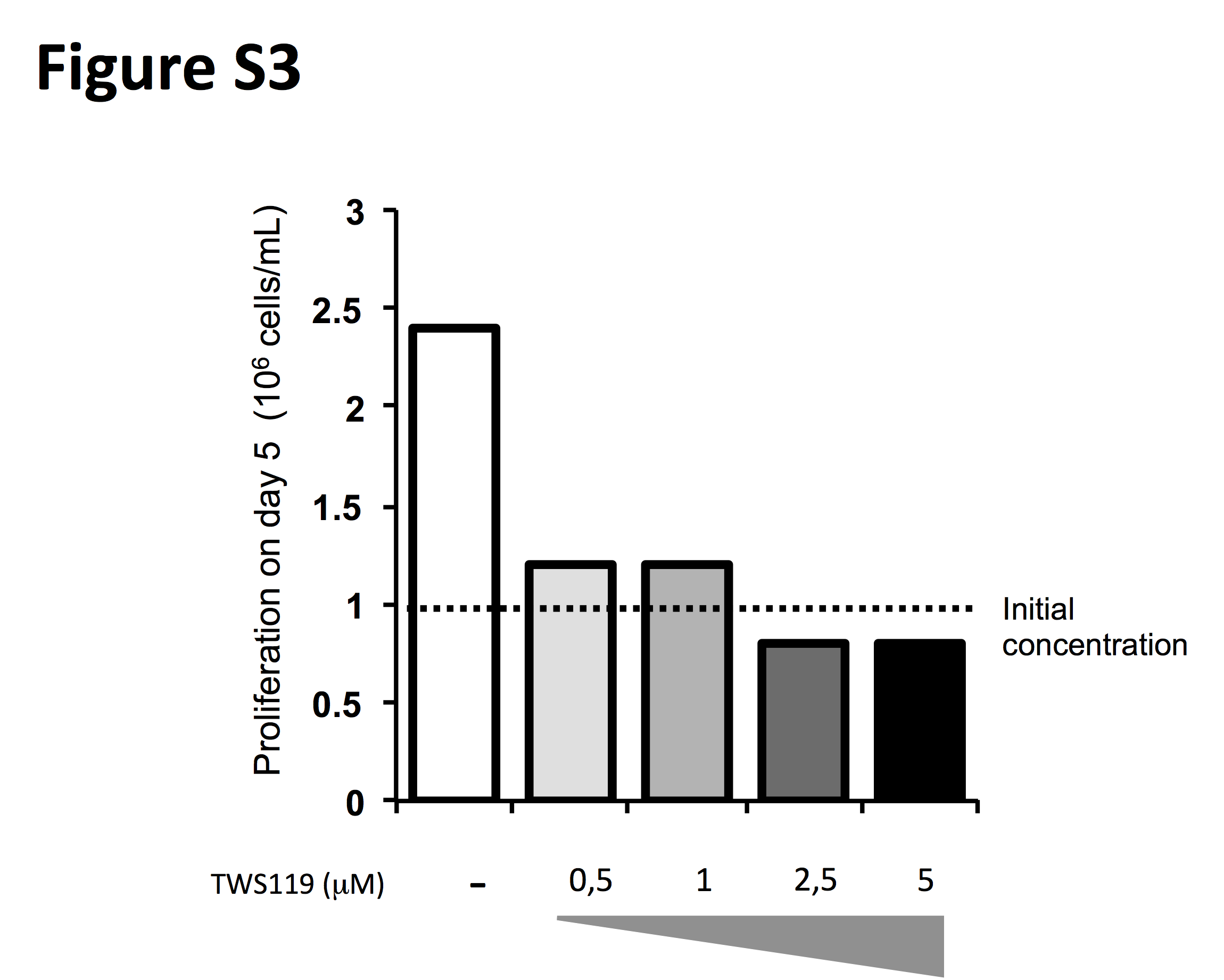

Supplement: Figure S3 — Proliferation of CD8+ T cells activated with anti-CD3/IL-2 following five days of culture with a TWS119 dose-response (0.5 μM to 5 μM), illustrated by final cell concentration. Cell counts were performed manually and viability evaluated by exclusion of trypan blue. (TIFF) [file pone.0041074.s003.tiff]

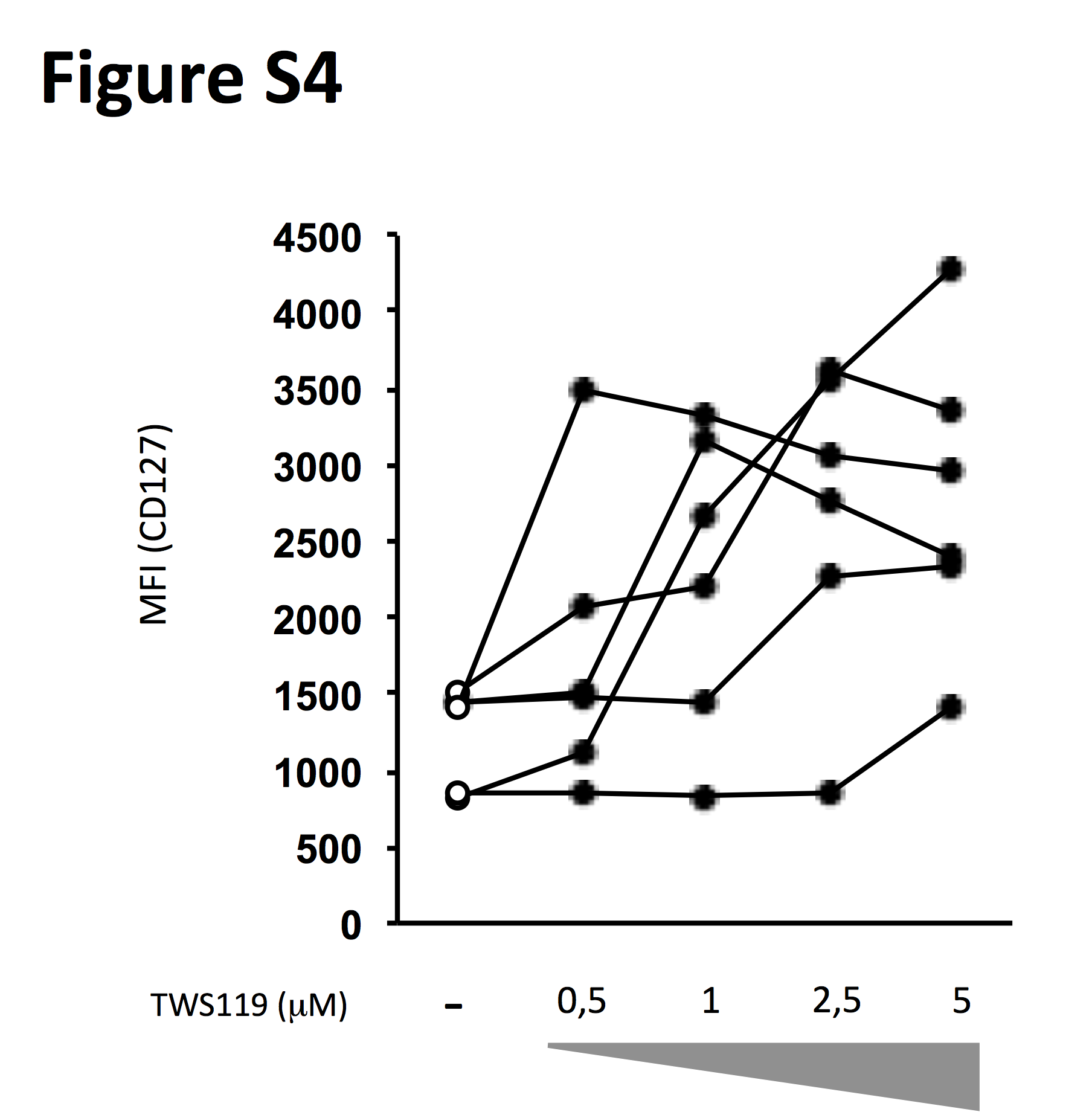

Supplement: Figure S4 — Evaluation of CD127 MFI of total CD8+ T cells activated with anti-CD3 and IL-2 for five days with a TWS119 dose-response (0.5 to 5 μM). Eleven HD were evaluated for CD127 and 5 HD for CD133. (TIFF) [file pone.0041074.s004.tiff]

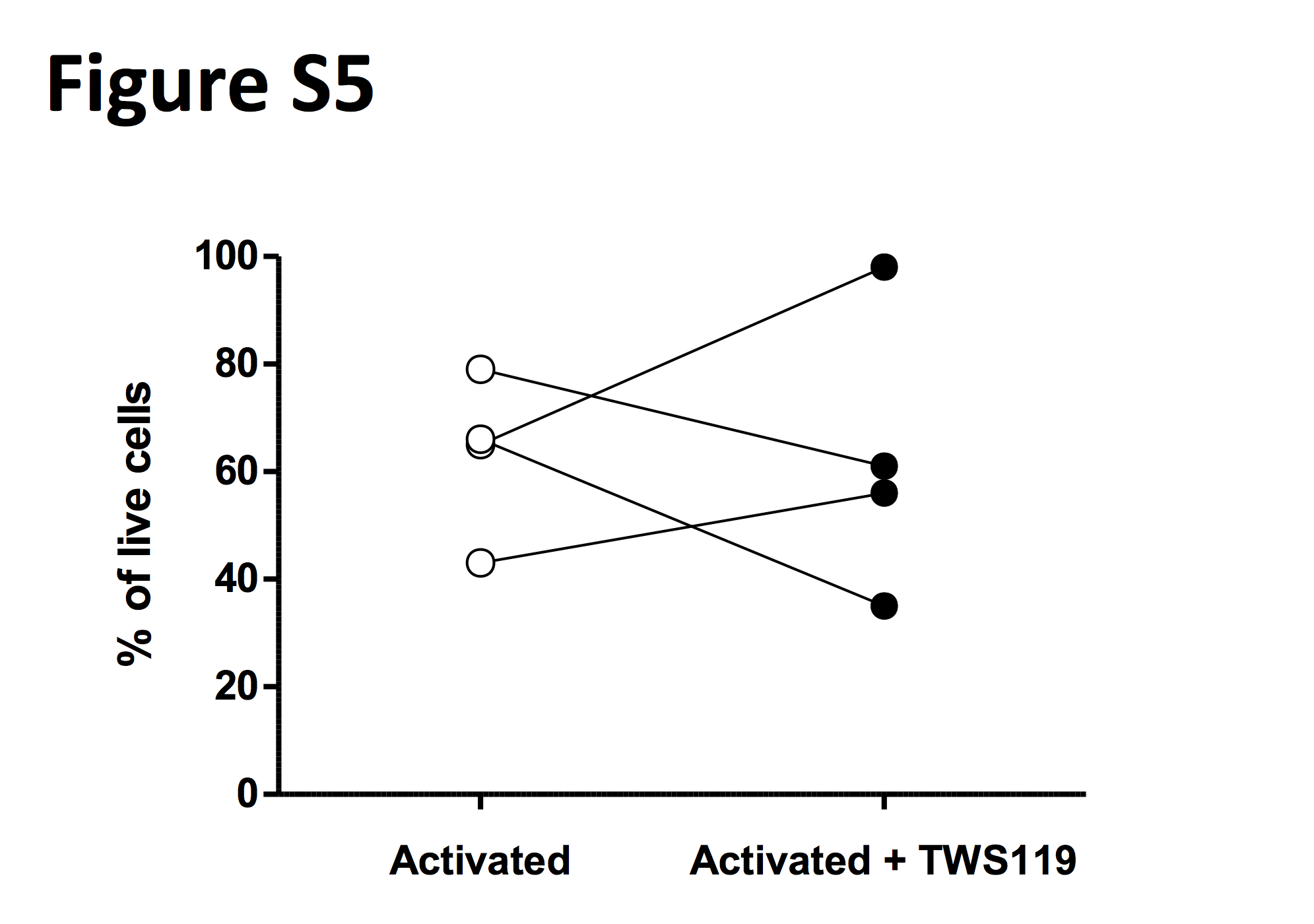

Supplement: Figure S5 — Percentage of live CD8+ TIL from lung cancer activated with anti-CD3/IL-2 after five days of culture with or without TWS119. Cell viability was evaluated by flow cytometry by gating on cells that excluded the viability dye (LIVE/DEAD® Fixable Aqua Dead Cell Stain Kit, Invitrogen). (TIFF) [file pone.0041074.s005.tiff]
